# The effectiveness and cost-effectiveness of a mindfulness training programme in schools compared with normal school provision (MYRIAD): study protocol for a randomised controlled trial

**DOI:** 10.1186/s13063-017-1917-4

**Published:** 2017-04-26

**Authors:** Willem Kuyken, Elizabeth Nuthall, Sarah Byford, Catherine Crane, Tim Dalgleish, Tamsin Ford, Mark T. Greenberg, Obioha C. Ukoumunne, Russell M. Viner, J. Mark G. Williams, the MYRIAD team, Daniel Brett, Liz Lord, Lucy Palmer, Anna Sonley, Laura Taylor, Anam Raja, Triona Casey

**Affiliations:** 1 Department of Psychiatry, University of Oxford, Oxford, UK; 2 Kings Health Economics, Kings College London, London, UK; 3 MRC Cognition and Brain Sciences Unit, Cambridge, UK; 4 University of Exeter Medical School, Exeter, UK; 5 Penn State University, Old Main, State College, PA USA; 6 NIHR CLAHRC South West Peninsula (PenCLAHRC), University of Exeter Medical School, Exeter, UK; 7 UCL Great Ormond St. Institute of Child Health, London, UK

**Keywords:** Adolescence, Schools, Resilience, Mindfulness, Depression, Prevention

## Abstract

**Background:**

Mindfulness-based approaches for adults are effective at enhancing mental health, but few controlled trials have evaluated their effectiveness or cost-effectiveness for young people. The primary aim of this trial is to evaluate the effectiveness and cost-effectiveness of a mindfulness training (MT) programme to enhance mental health, wellbeing and social-emotional behavioural functioning in adolescence.

**Methods/design:**

To address this aim, the design will be a superiority, cluster randomised controlled, parallel-group trial in which schools offering social and emotional provision in line with good practice (Formby et al., Personal, Social, Health and Economic (PSHE) Education: A mapping study of the prevalent models of delivery and their effectiveness, 2010; OFSTED, Not Yet Good Enough: Personal, Social, Health and Economic Education in schools, 2013) will be randomised to either continue this provision (control) or include MT in this provision (intervention). The study will recruit and randomise 76 schools (clusters) and 5700 school students aged 12 to 14 years, followed up for 2 years.

**Discussion:**

The study will contribute to establishing if MT is an effective and cost-effective approach to promoting mental health in adolescence.

**Trials registration:**

International Standard Randomised Controlled Trials, identifier: ISRCTN86619085. Registered on 3 June 2016.

**Electronic supplementary material:**

The online version of this article (doi:10.1186/s13063-017-1917-4) contains supplementary material, which is available to authorized users.

## Background

In the UK, the annual economic cost of mental health problems has been estimated at £105 billion [1, 2]. Mental health problems commonly have their first onset in adolescence, which is a period of heightened vulnerability associated with reduced attentional, emotional and behavioural regulation in the face of growing demands [3, 4]. In fact, 50% of adults with psychiatric disorders experience clinically impairing psychopathology before age 15 years, and 75% by age 24 years [5].

Of all mental health disorders that emerge during adolescence, depression is the one with the largest impact on health throughout the lifespan in terms of Years Lost to Disability [6]. Among adults with recurrent depression, the earlier their depression first develops, the more severe its subsequent clinical course [7]. Onset in childhood or adolescence is associated with greater impairments in social and occupational functioning and reduced quality of life, with adolescent depression associated with poor academic performance, family and social difficulties, physical ill-health, suicide attempts and completed suicide [8–11]. Such increased severity of early onset depression is also reflected in the fact that within child and adolescent samples, depression is often comorbid with other disorders; more than a third of these young people have a disruptive behavioural disorder, anxiety disorder or both [12, 13]. It is, therefore, vital that effective interventions are developed to tackle these vulnerability processes and to target those interventions during this critical window of adolescence.

There have been many calls to develop programmes for adolescents to reduce risk of mental ill-health, promote wellbeing and develop life skills across the spectrum of wellbeing and functioning [14, 15]. Because of their broad reach and central role in the lives of children and families, schools are seen as the primary setting where such efforts should be focussed [16]. However, there are many challenges to implementing such school-based programmes. In particular, targeted interventions, selectively offered only to adolescents deemed at risk of later mental health problems, face substantial costs associated with screening and can be stigmatising. Critically, they also miss those currently deemed at lower risk, but whose risk profile changes later.

As an alternative, recent systematic reviews and governmental reports suggest that school-based universal approaches, offered to the whole population, have the most potential to promote the mental health of young people [17, 18, 19]. However, the current research highlights that for such universal interventions to succeed, several key pragmatic and theoretical issues need to be considered [20]. At the pragmatic level, many programmes do not consider fully how best to support teachers to deliver the intervention competently [18] or try to implement programmes without due attention to known implementation facilitators and barriers [21].

Even if these pragmatic concerns are resolved, more fundamental theoretical issues still remain. Many existing universal interventions, aimed at reducing the risk of depression in young people, are based on theoretical models originally developed to address established psychopathology (e.g. cognitive theory and therapy) *– that is, they are designed to be used when people are unwell*. They therefore lack relevance, both for low-risk adolescents and for those who are at high risk, but not currently showing symptoms. To illustrate, a recent UK, fully powered, large-scale, cluster randomised controlled trial (RCT) based on cognitive behavioural therapy (CBT) principles had good reach, but low acceptability [22]. The study found that the intervention had no effect compared to usual school provision or attention control conditions [22], consistent with other recent well-designed RCTs [14, 23, 24].

This suggests that the theoretical basis for an effective universal intervention needs to focus on those critical psychological mechanisms that are universally relevant for the whole spectrum of mental health, from risk at one end, through resilience, to flourishing at the other end. The proposed trial is grounded in such a framework and evaluates a method of mental training (*mindfulness*) to modify these core mechanisms which can be used by all young people.

Our key theoretical premise is that mental health and wellbeing are compromised when there is diminished ability to effectively harness top-down executive control to pursue goals and plans when faced with salient, competing distraction from bottom-up processes [25–29]. The significance of this premise is that this proposed central cognitive mechanism applies not only to those at risk, but also across the wellbeing spectrum.

For individuals at risk of *internalising* problems, such as depression and anxiety, deficits in executive control manifest as difficulty in regulating cognition, affect and behaviour in the face of distracting, intrusive, negative thoughts and feelings [30–36]. For those at risk of *externalising* problems (conduct and antisocial/disruptive behaviour), deficits in executive control manifest as impaired impulse regulation, a problem that is associated with long-term impairments across multiple domains of functioning [37, 38].

For those who are *resilient or flourishing*, executive control enables the effective deployment of attention in the face of relatively innocuous, but habitual, patterns of thought (e.g. rumination) that can distract from current plans, exacerbate everyday stresses (affecting test-taking, sports performance and sleep) and undermine wellbeing [39, 40]. In sum, the hypothesis is that enhancing executive control in the face of these diverse challenges will both reduce risk for vulnerable adolescents as well as promote flourishing among those who are already resilient.

Our theoretical framework points us towards a training method that focusses on modifying key executive processes, instead of focussing on reducing pathology-specific negative patterns of thinking and behaviour. Our programme aims to examine one such method, mindfulness training (MT) which is specifically designed to address such processes [29] and can be used when people are well [41, 42].

MT involves systematic practice in focussing attention in a sustained and intentional way. It augments the ability to exercise top-down executive control in the face of motivationally compelling distractions [43–46]. It also reduces intrusive thoughts and ensuing ruminative responses [47–50] and behavioural impulses [51]. MT has been developed as a preventive intervention for those who already have enduring mental health problems. For example, mindfulness-based cognitive therapy (MBCT) was developed for people with a depression history but who are currently well, to prevent future depressive relapse [52]. The evidence base for its effectiveness and cost-effectiveness is growing [53–55], and it is now recommended by National Institute for Health and Care Excellence (NICE) (2009) as a first-line psychosocial treatment for secondary prevention of recurrent depression. Early studies suggest that MBCT’s preventive effect was greatest in those who had experienced three or more prior episodes. However, we now know that the number of episodes predicts good response because it is a marker for those with greater vulnerability due to pre-adult onset of depression and early adversity [56]. The effects of MT are not, however, confined to vulnerable groups. It has been found to have beneficial effects, via executive function changes, in nonclinical populations [47, 57]. This suggests that MT is not only acceptable to nonclinical populations, but also has huge promise for *primary* prevention of depression because it enables intervention in early adolescence, the point at which such vulnerability first emerges.

The research question is:

‘Does MT have the potential in adolescents to shift the population away from psychopathology and towards improved mental health and wellbeing by addressing key processes of mental regulation and executive control that operate across the spectrum of risk/resilience?’

The acceptability and feasibility of MT in young people appears promising [58–62]. However, there are many unanswered questions about its ability to prevent future depression and other mental health problems in adolescence, its mechanisms of action and what moderates its effectiveness. Also, there are no robust RCTs – grounded in theory and using an adequate follow-up period – that have evaluated the benefits of MT across the whole spectrum of risk/resilience in adolescence [60].

A prototype of a school-based MT programme has been developed by classroom teachers to teach mindfulness skills in a UK context as an integral part of the school curriculum [63]. This MT programme was piloted against matched comparison schools, including some schools with higher than average deprivation scores and more children with special needs. Not only was MT acceptable to secondary school children and teachers, but compared with normal school provision of social-emotional teaching, MT also reduced children’s depressive symptoms and increased their wellbeing. This was maintained at 3-month follow-up (adjusted mean differences: depression, (Center for Epidemiologic Studies for Depression Scale; CES-D [64]), −1.4, 95% CI −2.3 to −0.05, *p* = 0.005; wellbeing, (Warwick-Edinburgh Mental Wellbeing Scale; WEMWBS [65]), 3.0, 95% CI 0.0 to 6.0, *p* = 0.05). Effects on wellbeing and depressive symptoms were most marked at times of highest stress, and greater use of mindfulness skills was associated with stronger effects [66].

Provisional evidence from this nonrandomised feasibility trial is encouraging. Moreover, interventions that are designed with implementation in mind are likely to prove more acceptable [21, 67]. When adapted appropriately, MT is acceptable in more deprived and culturally diverse settings [68] and among young people with attention and conduct disorders [69]. Importantly, preliminary evidence suggests that MT in schools benefits not only young people, but also shows promise in enhancing teachers’ self-efficacy and wellbeing [58, 70, 71]. There is a need for an adequately powered RCT – that uses validated outcomes assessed over meaningful time frames – of a theory-based and thoughtfully implemented MT programme. Moreover, as the MT is delivered as a universal school intervention, a cluster RCT is required where schools are the units of allocation.

This study protocol describes a cluster RCT designed to evaluate the effectiveness and cost-effectiveness of including a MT programme within provision of social-emotional teaching compared with social-emotional TAU for young people aged 12–14 years within secondary schools. This protocol has been informed by learning from two feasibility studies [66, 72] and several large-scale school-based studies [12, 16, 67, 73–77]. The protocol is written in conjunction with the Standard Protocol Items: Recommendations for Interventional Trials (SPIRIT) guidance for protocols [78], see Additional file 1. The trial will comply with the Ottawa Statement on the ethical conduct and design of cluster RCTs [79] and the findings will be reported in accordance with the 2010 Consolidated Standards of Reporting Trials (CONSORT) Statement [80] (including its extension to cluster RCTs [81]).

The primary aim is to determine the effectiveness and cost-effectiveness of the MT programme on three co-primary self-report outcomes at 2-year follow-up, measured at the level of the individual young person:

1. Risk for depression,
2. Socioemotional and behavioural functioning, and
3. Wellbeing.

Broader secondary individual-level outcomes for students will include executive functioning, drug use, peer relationships, anxiety, attainment and mindfulness skills. Teachers will also rate the pupils on socioemotional and behavioural functioning.

For teachers, secondary outcomes will include stress, anxiety, depression, burnout and classroom mindfulness.

Secondary school-level outcomes will include school ecology and climate.

## Methods/design

### Study design

**Fig. 1:**
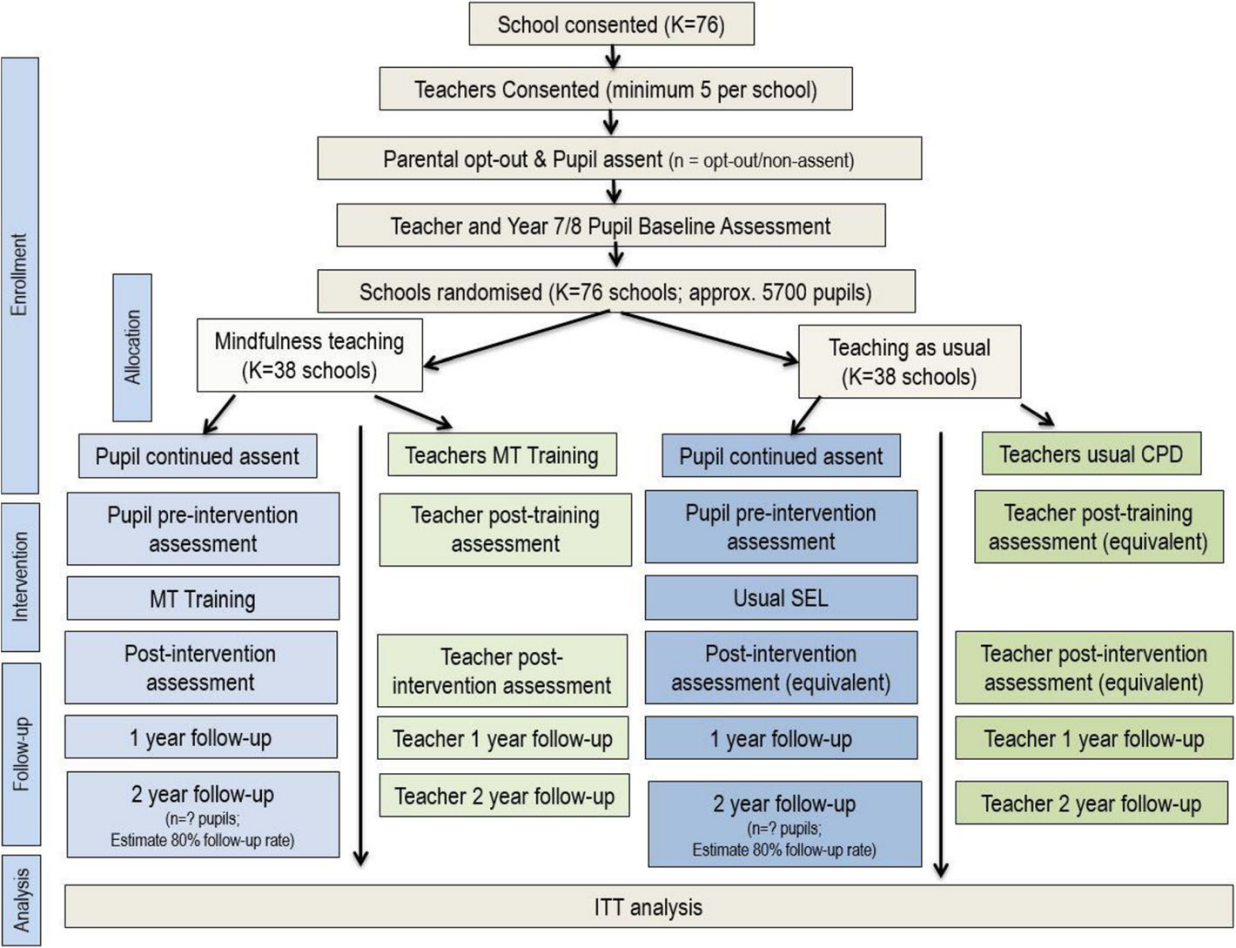
Consolidated Standards for Reporting Trials (CONSORT) diagram: participant flow, showing recruitment, training, intervention and assessment schedule

The design will be a superiority, cluster randomised controlled, parallel-group trial in which inclusion of the MT programme within school social-emotional teaching provision will be compared with provision of school social-emotional teaching as usual (teaching as usual, TAU), in 76 schools (clusters); 6840 school students (ages 12–14 years) will be approached to recruit 5700 (Fig. 1, CONSORT diagram). To ensure that baseline measures are collected for all clusters (i.e. schools) prior to randomisation pupils (approximately 25,000) will be enrolled into the study to provide baseline assessments (primary measures only), usually across both years 7 and 8. Only a subset of these pupils, who are members of classes subsequently selected to participate in the full trial, will move on to become full trial participants the following year. The reason for conducting baseline assessments with all pupils in the relevant year groups is that assignment of pupils to class groups varies in some schools from year to year. It is not, therefore, possible to randomly select and baseline pupils from a subset of classes at the outset to the trial and be confident that these pupils will still be taught together in the following, intervention, year. Conducting baseline assessments with all pupils ensures that these data are available for all pupils who might conceivably be grouped together in the year that the intervention will be delivered.

The definition of trial participants will be those who provide data at the baseline assessment and are members of one of the classes subsequently selected for continued trial participation. Those pupils who do not provide baseline assessment data (for example, due to absence) but are subsequently present in selected trial classes will not be included as trial participants.

A two-arm trial as opposed to a three-arm trial is employed for several reasons. First, the research question addresses the key remaining uncertainty: does MT add value over current UK good practice in relation to social-emotional teaching? Second, MT’s mechanisms of action, relative to an active control condition, are examined through a separate programme of work. Third, our experience is that cluster RCTs on this scale are most likely to be a rigorous test of effectiveness/cost-effectiveness when they are as simple as possible and when school heads, teachers and pupils perceive there to be equipoise between the two arms.

### Setting

Secondary schools chosen to be recruited are broadly representative of those UK secondary schools which offer social-emotional teaching in line with good practice guidance and are open to having the content and quality of their provision monitored.

### Participants and eligibility

A sample of mainstream UK secondary schools will be recruited that is representative of such schools, both with respect to the population served (on key variables such as deprivation, operationalised as eligibility for free school meals) and the type of school (e.g. selective/nonselective, urban/rural, large/small, mixed/single-gender, state maintained/independent). We will not include special schools or alternative settings where education is provided. Only schools that offer social-emotional teaching in line with good practice will be eligible for participation, determined using a measure designed for this study to benchmark against key dimensions. Schools must also be willing to commit to the My Resilience in Adolescence (MYRIAD) study, including the teacher training required in schools randomised to MT and teacher and pupil assessments and follow-ups. To mitigate risk to implementation, schools that are rated by national inspectors on measures of quality as ‘inadequate’, or where there is no substantive head, will be excluded.

Within schools, participating teachers will be qualified/experienced teachers who have given their consent to participate in the research and to complete the training in the delivery of the MT programme and subsequently to deliver the MT programme, should their school be randomised to the MT arm of the trial. Should the school be randomised to the TAU arm these teachers will be assessed as ‘controls’. They will normally be on substantive teaching contracts to increase the likelihood that they will remain teaching within the school during the research period.

### Recruitment

Recruitment of schools, teachers and pupils will occur in two recruitment cohorts with each new cohort starting at the beginning of a school year. For the first cohort the aim will be to enrol a relatively small number of schools (approximately 13), with the remainder recruited in the following cohort. The first cohort will serve to ensure that all the protocols (e.g. recruitment) are fit for purpose before proceeding to the second cohort. A variety of recruitment strategies will be employed; for example: newspaper articles, email or telephone calls with local authorities and attendance at events for school representatives.

Where schools decide to participate in the trial they will agree to offer the MT programme as part of the standard school curriculum. That is to say parental opt-out and child assent relates to the research (baseline and full RCT assessments) and not the MT or TAU. Consent to participation in MT or TAU curricula is at school level. Schools in the MT arm will be free to teach the MT programme to nontrial classes as they wish, but we will not collect data from these pupils.

### Randomisation procedure

Schools (clusters) will be randomised using computer-generated random numbers by an independent statistician. The schools will be assigned unique study numbers so their identities are unknown to the statistician, thus ensuring allocation concealment. The schools in each cohort will ideally be randomised as a single batch. The following are a selection of the variables that will be considered for stratifying the randomisation, with final decisions taken once schools have been recruited in each cohort: school size (large/small), type and quality of school (selective/nonselective, independent/nonindependent, mixed/single-gender, school-quality measure, e.g. OFSTED), geographic location (urban/rural and region) and level of deprivation (e.g. below or above median of children eligible for free school meals).

Participating children will normally be recruited from the schools in the autumn term of the school recruitment years (September through December) [82].

### Interventions

The MT programme and TAU will be delivered at school (cluster) level. Both will be mapped as far as possible using a template for intervention description and replication (TIDieR) checklist and guide for reporting complex interventions [83]. UK schools deliver social-emotional teaching in different ways and will likewise choose to integrate MT within their existing provision in different ways. Thus, the trial cannot be categorised as either purely additive (MT in addition to existing social-emotional teaching provision) or substitutive (MT replacing existing social-emotional teaching provision) in its design. Rather, different schools will be at different points on this hypothetical continuum and we will report details of provision of social-emotional teaching in schools across both arms of the trial, collected through a bespoke measure administered by the research team to one or more relevant staff members within each school and supplemented by review of school policy documents and other relevant materials.

### MT programme [63]

The MT goals are to enable adolescents to learn mindfulness skills that enhance mental regulation and executive control across the spectrum of risk/resilience. The MT programme is drawn primarily from MBCT [52]. A unique feature of the MT programme is its focus on the full spectrum of functioning from mental health problems to flourishing, enabling all young people to use mindfulness skills to manage emotions, academic study, sport, sleep and relationships. It was developed over more than 5 years by three classroom teachers (Richard Burnett, Chris Cullen and Chris O’Neill) who are also experienced mindfulness practitioners. This has included ensuring that the programme can be taught in mainstream schools, how best to engage hard-to-reach children and how to manage challenging classroom behaviour. It has been developed and adapted to ensure that it is acceptable to diverse school contexts and student populations. Latterly, the programme has been enhanced to support children to practice mindfulness during and beyond the course.

The MT programme comprises several elements, delivered through the school curriculum, over several years, supported by teacher training. The bulk of the MT programme is taught to students in a set of 10 structured lessons (within the trial, taught in years 8 and/or 9). The MT programme will normally be delivered in the spring terms (January through April), with support to continue use of mindfulness skills into the summer term. In the following school years, there are follow-on lessons intended to continue and support further learning and ongoing mindfulness practice (e.g. lunchtime clubs or drop-in sessions). This follow-on training in subsequent school years aims to sustain, deepen and begin to apply students’ learning; for example, to managing tests and examinations and to embed mindfulness in the school ecology/climate.

The MT programme includes a combination of psycho-education and practical skills involved in training the mind, learned in an experiential way, through short mindfulness practices which focus on the breath, body and immediate experience. There is also classroom discussion of the application of new skills in everyday life. Its design aligns with principles identified as important for effectiveness in several reviews of schools-based programmes that promote mental health and wellbeing and teach social and emotional competence. These principles include: explicitly teaching skills and attitudes; tailoring components and approaches to the needs of young people; using a range of age-appropriate, interactive, experiential and lively teaching methods; providing age-appropriate resources; for example, in this context resources that bring mindfulness to life (including a course booklet, a set of mindfulness exercises provided online and mindfulness practices that are introduced through animations and available as digital downloads); intensive, focussed teacher education to build teachers’ self-efficacy and wellbeing; and programme implementation which pays close attention to clarity and fidelity, in this case supported by a manual and indicative script [19, 21, 58]. Building on data that greater practice is associated with better outcomes [84, 85], the MT programme includes strategies to support teachers in keeping mindfulness integral to the culture of their year group/the school as a whole. Examples of good practice in this area could include teacher catch-up days/support events, suggested schedules for progressive, regular mindfulness input throughout year groups, suggested smartphone apps and using parts of the MT programme in core curriculum subjects.

Whilst all participating schools randomised to MT will have agreed to deliver the MT programme to a minimum of three classes within years 8 and/or 9, they will be encouraged to consider how they might introduce mindfulness into the curriculum more broadly, for the potential benefit of other school pupils and the wider school climate.

Because implementation affects both reach and outcomes [86], all schools will be supported with implementation guidance to increase the likelihood that MT is introduced into the schools in ways that maintain its integrity and are sustainable. For example, implementation of MT will require engagement with school leadership teams, teachers and pupils, as identified; for example, in research in disadvantaged urban schools in the US [77, 87].

### Training teachers to deliver the MT programme

The training programme to deliver the MT programme involves teachers first participating in an 8-week MBCT programme, adapted for the general (nonclinical) population, to support the development of their resilience and mindfulness skills (eight 2-h sessions per week, with an all-day mindfulness session supported by a digital app to facilitate mindfulness practice during and after the 8-week course). They will then attend a 4-day training workshop to learn how to deliver the MT in schools, with support where needed when they move onto teach themselves. Within participating schools, as large a pool of teachers as possible will be trained to build in redundancy should teachers either not achieve required levels of competency or leave the school. Training a larger group of teachers will also support greater implementation of the MT programme within the school, outside trial classes, as well as offering peer support throughout the project.

#### Fidelity of the MT programme

To test the effectiveness of the MT programme we need to ensure that it is delivered with fidelity. The teacher training and MT programmes are highly structured and standardised to maximise fidelity. Through teacher selection and teacher training we will endeavour, as far as possible, to ensure that teachers reach an adequate standard before they teach trial classes. During the trial classes, competency/adherence will be monitored. Independent raters will rate a randomly selected subset of videotapes of MT programme classes for fidelity (adherence and competence) using a standardised measure developed by the study team with adaptations made for MT in schools [88, 89].

### Teaching as usual in line with good practice

The trial aim is to establish if MT, when integrated into social-emotional teaching in secondary schools, adds value over and above current good practice. Recent UK Department of Education reports suggest that 60% of secondary schools offer Personal, Social, Health and Economic Education (PSHE) lessons that are ‘good or better’ and that this provision occurs across ages 11–16 years (Key Stages 3 and 4) through a variety of methods including regular scheduled lessons, drop-down days, within other subjects, and in tutor/form time [17, 90]. Determining whether schools have good PSHE provision is challenging. In cohort 1, schools will be eligible for inclusion if their provision of PSHE (or equivalent) meets four criteria: (1) the presence of discrete, regular, named teaching time for PSHE, (2) a named PSHE lead, (3) a written PSHE policy and (4) a named member of the senior leadership team responsible for PSHE. TAU schools will agree not to provide the MT programme (or other curricula that include MT) until study completion. We will conduct a more detailed enquiry into the provision of PSHE in general and social and emotional learning (SEL) elements, in particular at each of the cohort-1 schools (see below), and will use the findings of this to describe provision and modify inclusion criteria for cohort 2 if required. This approach ensures that MT’s effectiveness is tested against current good practice.

### PSHE/social-emotional teaching as usual in both trial arms

Following randomisation, the current provision of social-emotional teaching will be explored using a bespoke tool developed for the MYRIAD trial drawing in part on existing measures [17]. This will enable us to report upon how schools describe their current practice with respect to PSHE in general, and social-emotional teaching in particular, in all randomised schools at the school level and for study pupils within each school and across both trial arms. It will further provide information on how the MT curriculum is integrated into wider teaching provision in intervention schools. This measure will be used initially in cohort 1 and both it, and the initial PSHE eligibility criteria, will be modified if necessary for subsequent cohorts.

### Baseline assessment and follow-ups

**Fig. 2:**
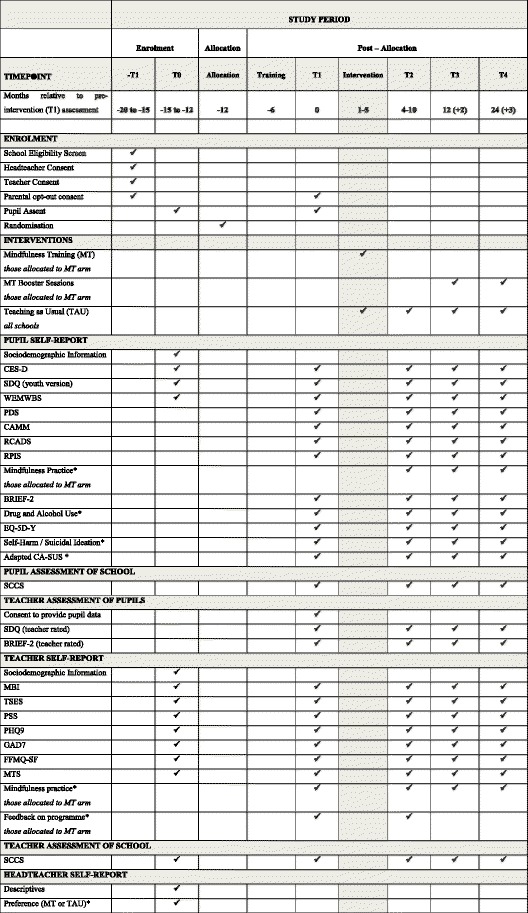
Standard Protocol Items: Recommendations for Interventional Trials (SPIRIT) diagram detailing trial activities and measures and their timing

Study outcomes will be measured at school consent/baseline (prior to randomisation^1^), preintervention (school term before intervention begins or equivalent), post intervention (within 3 months of the end of the MT programme or equivalent time in the TAU arm), 1-year follow-up (1 year after preintervention measures) and again at 2-year follow-up (2 years after preintervention measures, see Fig. 2). The data gathered from study participants and other sources at each time point are shown in Fig. 2.

### Sample size

The study requires 76 schools in total. All year-7 and year-8 pupils in each participating school (approximately 25,000 in total) will be invited to take part in a baseline assessment. In the subsequent academic year pupils who are members of three or more randomly selected classes (approximately 90 pupils in total per school) will be eligible to participate in the full trial. Thus, approximately 6840 pupils will be eligible to participate in the full trial with the expectation that 5700 will have provided parental consent/pupil assent and 4560 will ultimately complete the trial at 2-year follow-up. Drawing on the two feasibility studies [66, 72], a conservative assumption is made that in each class of 30, 25 children will have consent/assent to participate, and 20 of these will be followed up over 2 years. However, because parental opt-out consent and pupil assent will have been obtained prior to trial class selection where levels of consent/assent are lower than expected, we will have the flexibility to include more classes within a school in order to ensure that the required number of participants (75 in each school) proceed to full trial participation. The 38 schools (clusters) and 2280 children in each trial arm at follow-up (76 schools and 4560 children altogether) is a large enough sample to detect a difference of 0.2 standard deviation units (effect size) on our continuous co-primary outcomes. The sample size has been inflated to allow for multiple testing, setting the two-tailed significance level (alpha) for comparing each individual outcome between the trial arms to 0.0167 to preserve the overall family wide Type I error rate at 0.05. The study has 90% power to detect the specified effect size for each co-primary outcome as statistically significant. The sample size also allows for: (1) clustering of outcomes within schools, assuming an intracluster (intraschool) correlation coefficient (ICC) of 0.04, and (2) 20% dropout, with 60 of the 75 children consenting to participate in the trial providing full follow-up data within each school. Relevant literature suggests that our assumed value of the ICC is conservative. The largest ICC in one of our feasibility studies [66] was 0.037. The ICC for the same measure of depression as used here (CES-D) has been estimated to be 0.033 from a previous study in Quebec based on around 5000 children from across 71 schools drawn from relatively disadvantaged communities [91] and to be 0.009, 0.015 and 0.017 for different year levels (year 8, year 9 and year 10, respectively) based on around 2500 children from across 25 state-funded schools in South Australia spanning the full socioeconomic spectrum [92].

### Outcome measures

Multimethod and multi-informant measures will be used that have reliability, validity and established sensitivity to change, balanced with consideration to minimising burden on both participants and researchers and maximising data quality. All measures will be completed either on paper or via an online system. The pupils will complete the measures in a classroom setting where possible. Details of the measures and the time points at which they are collected are shown in Fig. 2. Where pupils are absent from school on the day at which data are obtained we will liaise with relevant school personnel to determine the most appropriate way of gathering these data, and will endeavour to gather data from as many absent pupils as possible to maximise data completeness.

### Primary outcomes (pupil completed)

Our aim is to determine the effectiveness of the MT programme based on three *co-primary* outcomes at 2-year follow-up: *risk for depression* (Center for Epidemiologic Studies for Depression Scale; CES-D; [64]); *social/emotional/behavioural functioning* (Strengths and Difficulties Questionnaire, youth version; SDQ [93]); and *wellbeing* (Warwick-Edinburgh Mental Wellbeing Scale; WEMWBS [65]). There was consideration of selecting just one primary outcome but the research team, experts in the field who were consulted and peer review concluded that all three co-primary outcomes are critically important. The research team also considered combining the three co-primaries into a composite outcome. However, as the research question includes the specific effect of the intervention on each of the three aspects, not just the overall effect, it was decided to retain the primary outcomes in their natural form. Composite measures can obscure variation that would convey interesting and important information in our proposed work [94].

There are a number of reasons for the choice of primary outcomes. First, adult depression (like mental health generally) is predicted by a range of difficulties in adolescence, including not only low-grade depressive symptomatology, but also social/emotional/behavioural functioning [13, 95–97]. Second, MT is a complex intervention that is specifically designed for young people along the full spectrum of risk/resilience and mental health. The outcome measures, therefore, needed to assess both problems (e.g. depressive symptoms) and also positive mental health. In such instances, and in line with the Medical Research Council (MRC) Complex Interventions Framework [98] and evolving guidance in the literature [99, 100], a number of critical outcomes were chosen as co-primary outcomes that: (1) are targeted by MT, (2) cover the full spectrum of mental health risk/resilience and (3) predict later psychopathology/mental health.

### Secondary outcomes (pupil-, teacher- and school-based)

A range of individual-level *secondary outcome measures* have been chosen based on their value to education policy-makers, school heads and pupils themselves. Secondary outcomes are:

- Students’ executive functioning (Behaviour Rating Inventory of Executive Function, self- and teacher-rated versions; BRIEF-2 [101])
- Peer relationships (Resistance to Peer Influence Scale [102])
- Drug and alcohol use, assessed using a brief measure designed for the study
- Anxiety (anxiety subscales from the Revised Child Anxiety and Depression Scale; RCADS [103])
- Social, emotional and behavioural functioning (Strengths and Difficulties Questionnaire, teacher version [93])
- Student-level attainment (National Pupil Database [104, 105])
- Self-harm and suicidal ideation (measured devised for study)
- Mindfulness skills (Child-Adolescent Mindfulness Measure; CAMM [106])

To support resource allocation decision-making and guideline development by bodies, such as NICE [107], the EuroQol five dimensions measure of health-related quality of life, youth version (EQ-5D-Y) [108], suitable for the calculation of Quality-adjusted Life Years (QALYs) and application to economic evaluation, will also be included, alongside the Child and Adolescent Service Use Schedule (CA-SUS).

Given the high rates of teacher stress and burnout, the importance of school ecology/climate, and the potential of MT to address these variables, the following will also be secondary outcomes:

- Teachers’ wellbeing (Maslach Burnout Inventory, Educator Survey; MBI [109])
- Self-efficacy (Teacher’s Self-efficacy Scale; TSES [110])
- Personal mindfulness (Five Facet Mindfulness Questionnaire, short form; FFMQ-SF, [111])
- Teacher mindfulness (Mindfulness in Teaching Scale; MTS, [112])
- Stress (Perceived Stress Scale; PSS [113]),
- Depression (Patient Health Questionnaire; PHQ9 [114])
- Anxiety (Generalised Anxiety Disorder; GAD7 [115])

Teacher-level variables will be measured for those teachers within schools identified to teach the intervention prerandomisation, irrespective of which arm of the trial their school is subsequently randomised to. School (cluster)-level outcomes will include school ecology/climate (subscales most relevant to the intervention from the School Climate and Connectedness Survey (SCCS) [116]) and school-level attainment; for example, GSCE results (National Pupil Database).

Study outcomes will be measured at five time points: baseline (school and teacher as well as primary measures for all pupils from years 7 and 8); preintervention (school term before intervention or equivalent); 3 months post intervention (or equivalent); 1 year (1 year after preintervention) follow-up; and again at 2-year follow-up (2 years after preintervention). It is important that outcomes are measured over a short enough period to enhance data completeness as well as over a long enough period to examine emergent risk/resilience over time.

### Economic data

The economic evaluation will take a health and social care perspective, as preferred by NICE [107], but will additionally include education-based services, since evidence suggests that health and education make up the majority of the costs of caring for young people with depression [117].

Service use will be recorded using a brief version of the Child and Adolescent Service Use Schedule (CA-SUS), successfully applied in previous adolescent depression populations [117]. A brief version focussing on key services (high cost and high volume of use), suitable for self-completion by parents of primary school children, is currently being applied in a similar school-based cluster RCT [73]. This measure will be adapted for application to an older population and for self-completion by the young people. Economic data will be collected at pre and post intervention as well as at 1- and 2-year follow-up. The preintervention measure will collect information covering the previous 3 months; at follow-up the service use will be recorded for the period since the most recent prior assessment of this data .

Resource inputs into MT training and delivery will be recorded as part of the trial and will be costed using a micro-costing approach. This will involve calculation of the cost of all individual elements (teaching and training staff time, any supply teaching expenses, training and intervention materials, etc.), as well as relevant overheads (administration, managerial, capital, etc.) and adjustment for indirect time (non-face-to-face working time which cannot easily be allocated to specific individuals). All other services used will be costed by applying nationally applicable unit costs, including National Health Service reference costs for secondary care services, as well as published costs for primary care, social care and education services [118].

Outcomes for the economic evaluation will be measured using the youth version of the EQ-5D measure of health-related quality of life (EQ-5D-Y) [108], shown to be valid and responsive to change in adolescent populations [119].

### Analysis plan

Analyses will be conducted/supervised by the co-investigator trial statistician (Obioha Ukoumunne) and trial health economist (Sarah Byford) and reported following CONSORT standards, overseen by the Data Monitoring Committee (DMC) and documented in a full prespecified statistical analysis plan. Analyses will be conducted on an intention-to-treat basis, with participants analysed according to the trial arm that they were randomised to, using multiple imputation to ‘fill in’ missing data. Comparisons will also be made between the trial arms, based on those with complete data in a sensitivity analysis. All between-arm comparisons will be run first as crude (unadjusted) analyses and then adjusted for baseline prognostic factors, chosen a priori, but certainly including the factors used to stratify the randomisation and, where measured, the baseline score of the outcome variable. The adjusted analysis will be considered to be the main analysis.

The approach to evaluating the intervention emphasises estimation of the intervention effect (confidence intervals), rather than strictly hypothesis testing. In recognition of the multiple testing, we will use an adjusted critical level for significance testing of 0.0167 for each of the three primary outcomes at 2 years to maintain the overall Type I error rate at 0.05. The confidence intervals will not be adjusted for multiple comparisons. No adjustments will be made to the critical levels for testing the primary outcomes at the earlier follow-ups, nor the secondary outcomes, as these are more exploratory in nature. The study sets out to establish the superior effectiveness and cost-effectiveness of MT compared with TAU. As set out above, all the co-primary outcomes are deemed important in their own right, such that each will be reported independently.

The main reported clinical analysis will use the intention-to-treat principle. The definition of a trial participant will be those who provide data at the baseline assessment and are subsequently in the classes randomly selected for participation in the full trial. In ancillary exploratory analyses we will also examine whether the effectiveness of the intervention is greater for schools, teachers and pupils that adhere to the curriculum (i.e. engage with intervention and, in the case of pupils in the MT arm, use the mindfulness practices). Because adherence is likely to be associated with factors that impact on the outcomes, we will account for this confounding using instrumental variable methods [120].

All analyses will account for clustering within schools as this is a cluster-randomised design. Continuous outcomes will be compared using random effects (‘multilevel‘) linear regression and binary outcomes will be compared using marginal logistic regression models using Generalised Estimating Equations (GEEs) with information sandwich (‘robust’) estimates of standard error, specifying an exchangeable correlation structure within clusters. Continuous outcomes will be summarised for each trial arm using means and standard deviations and binary outcomes will be summarised for each trial arm using numbers and percentages.

We will use tests of interaction to explore potential moderators of outcome, including, but not exclusive to: school level deprivation (proportion of pupils eligible for free school meals); the children’s age/year group; baseline risk for depression; wellbeing; and strengths and difficulties (SDQ). The latter is particularly important, as it is key to engagement with MT. These analyses are exploratory and hypothesis-generating in nature [121]. We acknowledge the issue of multiple testing and the need to cautiously interpret significant findings that will require replication in subsequent studies to have credence. We also acknowledge the low statistical power of tests of interaction in comparison to the power for detecting main effects [122].

Whilst mechanisms of action and potential mediators are examined in detail in a separate programme of work, we will explore potential mechanism variables pre and post MT in both trial arms and key outcomes at 1- and 2-year follow-up. We will ask if the change in mechanisms is specific to MT, changes as a function of use of mindfulness skills, precedes changes in the outcomes, and explains changes in key outcomes at follow-up. We will examine whether such changes occur over and above changes in those outcomes from baseline to post treatment [123] and through moderated mediation explore what works for whom. Methods for the analysis of mediation using clustered data are in the infancy of their development [124]. We will keep abreast of ongoing methodological research in this area and these analyses will be both exploratory and hypothesis-generating in nature.

Cost-effectiveness will be assessed in terms of QALYs using the EQ-5D-Y. Secondary analyses will explore cost-effectiveness in terms of the three co-primary outcomes to assess the sensitivity of analyses to the alternative outcomes of interest. We will employ standard methods of analysis, including multiple imputation for missing data, adjustment for baseline prognostic factors in line with the clinical analyses, and standard parametric tests for differences in costs, with the robustness of the parametric tests confirmed using bias-corrected, nonparametric bootstrapping [125]. Cost-effectiveness will be assessed using the net benefit approach, with uncertainty explored through the presentation of cost-effectiveness acceptability curves [126]. A within-trial analysis will be undertaken at 2-year follow-up.

The lifestyle choices and behaviour of young people on the threshold of adulthood can lead to short- and long-term adverse outcomes that are expensive for society and damaging to themselves [127, 128]. To this end, longer-term outcomes and costs will be explored using decision analytic modelling [129]. The model will be populated using data from our ongoing programme of work, including trial data and research on teacher training models, as well as evidence from the literature and relevant longitudinal cohort databases. The most suitable modelling framework in which to carry out the analysis will be dependent upon the results of the RCT, and thus will be finalised at a later point. Markov modelling is likely to be the most appropriate for extrapolation over the longer term since it is able to deal with relatively complex care pathways. The cost-effectiveness model will be analysed using incremental analysis and probabilistic sensitivity analysis. The time period over which the model will be run will be determined after review of the literature, since data availability is the key limiting factor. These analyses are exploratory and hypothesis-generating in nature [121].

### Minimising bias

To maximise *generalisability*, we will actively recruit schools that are representative of the UK population, with particular, but not exclusive, attention to key variables, such as deprivation, operationalised as eligibility for free school meals and region and the type of school (e.g. selective/nonselective, urban/rural, large/small, mixed/single-gender, state maintained/independent). As recruitment progresses we will, as far as possible, monitor recruited schools and teaching staff within these schools in terms of their match to these variables, actively seeking schools with characteristics that will improve the representativeness of the sample. In the event that we have more interested schools than we are able to recruit, we will make decisions on suitability based partly on the intention of achieving a representative sample of schools.

To minimise *contamination across clusters* we will randomise at the level of school, and secure schools’ agreement to adhere to the regime of the trial arm to which they are allocated. *Attrition bias* will be minimised by building on robust trial procedures developed in our feasibility trials [66, 130]. Retention of pupils is predicted to be 80% at follow-up. We have demonstrated that we can achieve close to 97% data from pupils and 100% retention of schools/teachers completion in our feasibility studies, albeit it with shorter follow-ups [66, 130]. As randomisation is at the level of school, if teachers leave, provision can be made within schools for cover by allocating another teacher able to offer the interventions. We will exclude schools from the study with an inadequate school quality rating or without a substantive head because of the risk to implementation. Trial newsletters and social networking will be used as a way of keeping in touch with schools and participants between follow-up points.

Robust randomisation procedures conducted by an independent statistician and prepublication of the trial protocol, and subsequently the data analysis plan, will minimise *subversion bias*. To limit potential for bias when unblinded researchers are involved in collection of self-report data from pupils, all researchers will be trained to introduce the study and measures in a standard way and provide standard responses to queries about the interpretation of questionnaire items.

#### Blinding of data files

The trial data file will be cleaned, locked and signed-off by the Trial Steering and Data Monitoring Committees before the trial statistician is unblinded. The remainder of the trial team will only have access to the unblinded data file following completion of main trial data analyses and presentation of main trial outcomes. Co-investigators other than Professor Willem Kuyken will remain blind to which trial arm each individual school is randomised as far as possible. Other members of the research team will be aware of school trial arm to facilitate implementation of the protocol within trial schools.

To *maximise data completeness*, data will be collected either through an online portal using tablets/laptops or through paper and pencil measures, whichever is preferred by the school/teacher/pupil. Pupils who are absent from school will normally be contacted through their school. In cases where pupils or teachers leave the schools, attempts will be made to follow them up to complete remaining measures. Teachers will be remunerated for completion of student-focussed measures. Time windows for the follow-up assessments will be large enough to maximise data completeness.

*Data management and integrity* will be maximised by using protocols established in our previous trials including using online data entry and, where appropriate, through double entry. All data will be stored securely in line with our data management protocol in order to protect the confidentiality of participants. Finally, *analytical biases* will be minimised by prepublishing the study protocol prior to randomisation and the statistical analysis plan prior to analysis.

#### Measurement of preference

In line with guidance for the design of RCTs [131], we will write our study materials to ensure that they provide clear information about the two trial arms. We will assess headteachers’ preferences at baseline.

### Trial governance

The management structure will ensure that the scientific aims are delivered and provide robust governance and oversight. Oxford University will sponsor and host the study and we sought ethics approval from the University of Oxford Central Research Ethics Committee.

A Trial Management Group (TMG) comprising the co-investigators and trial manager will provide day-to-day management of the project. TMG meetings will review progress against study milestones, plan work, discuss methods, keep a risk register and anticipate/resolve any problems. The first meeting will be face-to-face and then via video/teleconferencing throughout the project, with face-to-face meetings at least once a year. They will seek input from collaborators and others, as needed.

A Trial Steering Committee (TSC) and Data Monitoring Committee (DMC) will be established to provide trial oversight. They will be independent of the study team and trial sponsor and free of competing interests. The committees will be chaired and constituted by people with the requisite specialist expertise and experience. Copies of the TSC and DMC charters can be obtained from the authors on request. The Peninsula Clinical Trials Unit will support the trial in terms of database development, randomisation, and data management.

### Ethics

The investigator will ensure that this study is conducted in accordance with the principles of the Declaration of Helsinki. Ethics procedures build on our feasibility trials and other schools-based trials [73]. We will ensure consent at the school level from headteachers. We will then seek parental/caregiver opt-out and child assent. Consent from headteachers will be obtained electronically following detailed discussions between the headteacher or their representative and the research team. Consent from teachers will also be obtained electronically following a similar process. Parental opt-out consent will be managed by schools using ethically approved parent/carer and Pupil Information Sheets and an Opt-out Form and adopting the methods usually employed by the school for obtaining parental consent (e.g. electronic, hard copy or both). Pupils will provide assent at the start of the baseline assessment, through the computer terminals on which they will subsequently complete study measures, or occasionally, on paper. Cluster RCTs present particular ethical issues and we will, therefore, follow the Ottawa Group 15 consensus recommendations for cluster RCTs, with school headteachers identified as the ‘gatekeepers’ [79]. Child welfare and safeguarding procedures have been developed with input through our stakeholders (headteachers, teachers and young people). The investigators will ensure that this study is conducted in accordance with relevant regulations and with Good Clinical Practice. Researchers who will be obtaining informed consent will complete relevant components of Good Clinical Practice training. All members of the research team will undergo clearance through the UK disclosure and barring service. The study has received approval from the University of Oxford Central University Research Ethics Committee (CUREC). Any substantial change to the protocol design that alters the ethical frame of the project will be sent to the Ethical Committee for further review and any changes made as a result of this would be reported to the ISRCTN Registry. A random sample of approved CUREC projects may be monitored each year by the relevant CUREC subcommittee to review whether the research is being (or was) conducted within the scope of the ethical approval granted.

The recruitment and research governance procedures developed in the STARS (Supporting Teachers And childRen in Schools) trial [73] and our feasibility study will be used [66]. Our feasibility study did not identify any risks to young people arising from the research procedures or MT itself. However, a risk management protocol has been developed to provide a consistent approach to the identification and reporting of risk. This protocol will be discussed and agreed with the headteachers/safeguarding leads at each participating school and builds on the protocol developed in our earlier trials. The protocol will ensure that, where young people are identified as at risk of abuse, appropriate safeguards are put in place in a timely way. Young people who disclose concerns directly to the research team in person, or via another means of direct communication rather than through Case Report Forms, will be followed up to ensure that they receive appropriate support. All young people will be provided with bespoke information on local and national sources of support, the content of which will be agreed with participating schools. Likewise, participating teachers who are identified as at risk of harm will be followed up in accordance with the protocol for risk management. Data on serious adverse events (death, overnight hospitalisation, prolongation of existing hospitalisation, persistent or significant disability/incapacity, life-threatening situations and attendance at accident and emergency departments) will be collected as the research team becomes aware of them both as they arise and as part of routine data collection at each assessment point and will be reported to the DMEC within 7 days of the research team becoming aware of them. Adverse events will be logged and reported via the DMEC and TSC, and the DMEC will review aggregate data on child mental health and self-harm outcomes to ensure that there is no excess of such outcomes in the active arm. As we are collecting data in two cohorts, the end of the first cohort provides an opportunity for the DMEC and TSC to review these data and the robustness of these procedures once the first wave of intervention has been completed.

### Dissemination of outcomes

We are committed to maximising dissemination of knowledge arising from the MYRIAD trial and making the outputs of this work available to the widest possible audience. We will achieve this through the open access publication of research findings in high-quality peer review journals and through the appropriate presentation of the research at conferences and meetings. We will also endeavour to make our research directly available to relevant communities and groups, such as schools, teachers and young people, in an accessible format through a programme of public engagement activities planned in collaboration with our public engagement group. Details of plans for dissemination and authorship eligibility guidelines are outlined in the MYRIAD dissemination protocol v6, 14.11.15.

## Discussion

This cluster RCT aims to provide a rigorous evaluation of the effectiveness and cost-effectiveness of a MT programme, compared with good practice teaching of social-emotional curricula, for young people aged 12–14 years within secondary schools. It will answer a question with significant public health implications; namely ‘can a universal school-based intervention, in this case MT, shift the population away from mental ill-health and towards improved mental health and wellbeing?’

If the trial suggests that MT is cost-effective, this could enable schools to offer a relatively low-cost, scalable intervention to improve young people’s short- and longer-term social, emotional and mental health outcomes. This would have implications in terms of preventing mental health problems before they can take root and become a lifelong recurring problem. Moreover, there are prospective studies suggesting that the executive control skills MT seeks to develop are associated with a range of long-term health, social and economic outcomes [37]. This study will examine whether integrating MT into social-emotional teaching as usual, when compared with continuing social-emotional teaching alone, positively affects these pupil outcomes at 2-year follow-up. To assess longer-term outcomes we plan to establish a cohort, to follow participants up into adulthood, linked to the National Pupil Database. There are also significant potential benefits for schools in terms of teacher mental health, wellbeing and functioning and school ecology/culture. Finally, alongside our other programmatic work, the trial will contribute to our understanding of for whom, and when, MT is best delivered, its mechanism of action, and the most scalable approach to training teachers to deliver the MT programme.

## Trial status

Recruitment of schools began in late June 2016, with recruitment of pupils beginning in September 2016. Randomisation for schools in cohort 1 will occur in December/January 2016–2017. Recruitment for cohort 2 will begin in January 2017 and continue throughout the year with randomisation for cohort 2 planned for December/January 2017–2018.

## Additional file

Additional file 1: Completed SPIRIT 2013 Checklist for the MYRIAD Trial. (DOC 121 kb)

## Abbreviations

BRIEF-2: Brief Inventory of Executive Function, Second Addition
CAMM: Child and Adolescent Mindfulness Measure
CA-SUS: Child and Adolescent Service Use Schedule
CBT: Cognitive behavioural therapy
CES-D: Center for Epidemiological Studies Depression Scale
CONSORT: Consolidated Standards for Reporting Trials
CUREC: University of Oxford Central University Research Ethics Committee
DMC: Data Monitoring Committee
EQ-5D-Y: EuroQol five dimensions measure of health-related quality of life, youth version
FFMQ-SF: Five Facet Mindfulness Questionnaire, short form
GAD7: Generalised Anxiety Disorder, 7-item measure
GCSE: General Certificate of Secondary Education
GEE: Generalised Estimating Equations
ICC: Intracluster correlation coefficient
ISRCTN: International Standard Registered Clinical/soCial sTudy Number
MBCT: Mindfulness-based cognitive therapy
MBI: Maslach Burnout Inventory, Educator Survey
MRC: Medical Research Council
MT: Mindfulness training
MTS: Mindfulness in Teaching Scale
MYRIAD: My Resilience in Adolescence
NICE: National Institute for Health Care and Excellence
OFSTED: Office for Standards in Education, Children’s Services and Skills
PDS: Pubertal Development Scale
PHQ9: Patient Health Questionnaire, 9-item version
PSHE: Personal, Social, Health and Economic Education
PSS: Perceived Stress Scale
QALYs: Quality-adjusted Life Years
RCT: Randomised controlled trial
RPIS: Resistance to Peer Influence Scale
SCCS: School Climate and Connectedness Survey, teacher and pupil versions
SDQ: Strengths and Difficulties Questionnaire
SEL: Social and Emotional Learning
SPIRIT: Standard Protocol Items: Recommendations for Interventional Trials
STARS: Supporting Teachers and Children in Schools
TAU: Teaching as usual
TIDieR: Template for Intervention Description and Replication
TMG: Trial Management Group
TSC: Trial Steering Committee
TSES: Teachers’ Sense of Efficacy Scale
UK: United Kingdom
WEMWBS: Warwick Edinburgh Mental Wellbeing Scale
Year 10: pupils aged 14–15 years
Year 7: pupils aged 11–12 years
Year 8: pupils aged 12–13 years
Year 9: pupils aged 13–14 years

## Footnotes

1 For pragmatic reasons at this time point only the primary outcome measures for the pupils will be measured.
